# Neurofuzzy c-Means Network-Based SCARA Robot for Head Gimbal Assembly (HGA) Circuit Inspection

**DOI:** 10.1155/2018/4952389

**Published:** 2018-12-02

**Authors:** Somyot Kiatwanidvilai, Rawinun Praserttaweelap

**Affiliations:** Department of Electrical Engineering, Faculty of Engineering, King Mongkut's Institute of Technology Ladkrabang, Ladkrabang, Bangkok, Thailand

## Abstract

Decision and control of SCARA robot in HGA (head gimbal assembly) inspection line is a very challenge issue in hard disk drive (HDD) manufacturing. The HGA circuit called slider FOS is a part of HDD which is used for reading and writing data inside the disk with a very small dimension, i.e., 45 × 64 *µ*m. Accuracy plays an important role in this inspection, and classification of defects is very crucial to assign the action of the SCARA robot. The robot can move the inspected parts into the corresponding boxes, which are divided into 5 groups and those are “Good,” “Bridging,” “Missing,” “Burn,” and “No connection.” A general image processing technique, blob analysis, in conjunction with neurofuzzy c-means (NFC) clustering with branch and bound (BNB) technique to find the best structure in all possible candidates was proposed to increase the performance of the entire robotics system. The results from two clustering techniques which are K-means, Kohonen network, and neurofuzzy c-means were investigated to show the effectiveness of the proposed algorithm. Training results from the 30x microscope inspection with 300 samples show that the best accuracy for clustering is 99.67% achieved from the NFC clustering with the following features: area, moment of inertia, and perimeter, and the testing results show 92.21% accuracy for the conventional Kohonen network. The results exhibit the improvement on the clustering when the neural network was applied. This application is one of the progresses in neurorobotics in industrial applications. This system has been implemented successfully in the HDD production line at Seagate Technology (Thailand) Co. Ltd.

## 1. Introduction

Robotics and AI especially in neurorobotics play an important role in a number of manufacturing processes because of their fast processing time, good accuracy, intelligence, and high repeatability. In contrary, in the case of manual operation, users must have enough knowledge and experience for working with the processes. The manual operation typically results in inconsistency and cannot control the variations in accuracy and repeatability. Currently, HDD manufacturing processes are under development to be fully autonomous process by the implementation of Artificial Intelligence (AI) into the automation machine to replace the manual process from human. One of the most difficult processes is the visual inspection that has long been performed by experienced operators. The inspection requires many techniques since the human can naturally deal with the complex problem better than the machine. In addition, the decision of inspection based on the apparent image is still a challenging issue for the intelligent machine. To enhance the performance and accuracy of this process, AI techniques such as neural network, fuzzy system, and unsupervised learning are attempted to apply to the manufacturing, particularly in the visual inspection and control of robotics process. In this study, the HGA circuit inspection which is composed of visual inspection, SCARA robot, and classified boxes is developed. This process starts from the load-in of the incoming part, then the assembly process, and outgoing inspection using robot control. Before the development, the outgoing inspection inspects the FOS using the 30x microscope via the human eyes and then manually controls the robot to move the part into the corresponding box. This research aims to develop a neuro-fuzzy-based decision technique for this system to automatically control the SCARA robot for the HGA circuit inspection. Vision Pro program, which is a popular tool in image processing, is adopted as the platform of image processing in this study.

As stated in [1], blob detection is a simple but robust technique which was applied in many research studies such as field-programmable gate array with blob, fingertip blob recognition, and optimization [2, 3]. In our work, the simple blob is utilized as the tool for seeking the image features to apply in the next processes of classification and robot control. In general, the unsupervised learning on clustering techniques was widely adopted in many applications, and these were, for example, K-means clustering, K-means combined with PSO for document clustering [4], K-means and fuzzy c-means for document clustering [5], K-means applied on image clustering on the graphics processing unit (GPU) platform in [6]. As seen in [6], the developed platform could improve the processing time to be faster than the ordinary techniques. In [7], K-means clustering was applied to the map reduction framework, which is a huge data management to find the best value in the application. In [8], weighted least-squares model-based (WLSMB) with K-means was applied to enhance the ability of classification. Kohonen, which is one of the clustering techniques, has been proved in the application for the 3-dimensional data; in addition, the new model with iteration process could further help to improve the result [9]. The Kohonen map approach has been tested in [10] to solve the estimation problem, and the results showed a better performance than the basic belief assignment. One of the most popular clustering techniques is the fuzzy c-means. In terms of data envelopment analysis (DEA), the Kohonen neural network has been applied in [11]. In [12], fuzzy double c-means performed clustering well with different datasets on the data clustering and image segmentation. In medical area, the fuzzy c-means was applied for the magnetic resonance brain imaging [13], and experimental results exhibited the improved performance. In [14], the neurofuzzy c-means clustering algorithm showed the better robustness system on the experiment based on the synthetic datasets with suitable iteration. In the problem of software quality [15], the neurofuzzy c-means was applied to the fault prediction problem. Unsupervised and supervised data were tested on the training process in the model. Probability of detection is the key parameter for performance checking. However, the multiple clustering for new feature finding is required for future work. In terms of technology, the driver vigilance predictions on smartwatch-based driver or smart mobile device using neurofuzzy c-means were tested [16]. This research combined the measurement data from the sensor and clustering data for prediction. Neurofuzzy c-means clustering [17], which applies the Euclidean distance to the clustering technique, was utilized in many applications such as brain tumor in MRI images [18], remote sensing images [19], data clustering with image segmentation [20], and processing time improvement without performance effect [21]. In [22], branch and bound (BNB) was used for cyclic scheduling of timed Petri nets (TPN) based on the manufacturing systems. The speed of solving the block relocation problem [23] was improved by using the branch and bound algorithm. The concept is to minimize the number of necessary relocations; however, it still requires the future work to support the relaxed constraint. Also, BNB was applied in the model selection [24], the hand-eye calibration [25], the nonlinear integer programming with large-scale problem [26], the multiuser in wireless systems [27], and the maximum of weighted sum-rate for interfering links set [28]. All of the aforementioned techniques and applications were successful in the previous scopes. In this paper, a new improved classification technique for HGA circuit inspection using NFC and branch and bound technique are proposed to enhance the accuracy of inspection and increase the production rate. When the classification or groups of inspecting object has been decided, the action to move the SCARA robot will be the next process to transfer the object to the corresponding box.

## 2. Head Gimbal Assembly

**Figure 1:**
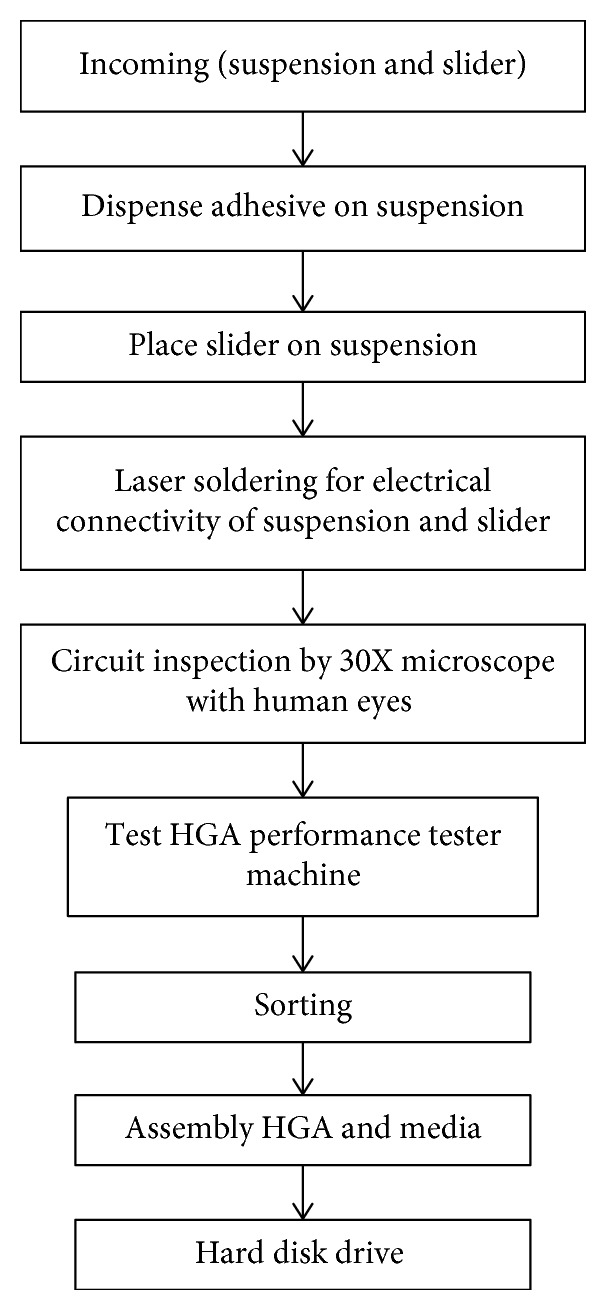
HDD manufacturing process flow.

Read and write processes of HDD are occurred from magnetic field changing on the disc. Inside the HDD, it may contain several discs which have a rotational speed during 7,200–15,000 rpm. The summary of the HDD manufacturing process flow is shown in Figure 1. As seen in this figure, the processes of the 30x microscope and slider placing on suspension are the main processes in HDD manufacturing. In addition, HGA is a part of HDD, which holds an electrical circuit inside the slider. Reader, writer, heater, temperature-activated circuits (TA), and microactuator are connected by the electrical circuits. Testing, sorting, and assembly process are the next steps to assemble the HGA stacks.

## 3. Image Processing and Feature Selection

In the automatic visual inspection system, there are two general sections needed to be considered and those are hardware selection and developing software for image processing techniques. In this study, a well design of image processing hardware has been considered and expected to provide good results for imaging. The field of view is a criterion to choose a camera, depending on the size of the slider and the depth of field (DOF) required. DOF is the minimum and maximum of the distance between the camera lens and the object that can yield clear image. Magnification is calculated by the ratio of camera working area and field of view as follows:

(1) $$\begin{array}{c} m=\frac{W_{\text{camera}}}{W_{\text{FOV}}}. \end{array}$$

Based on the careful consideration in selecting the hardware devices, camera, lens, and lighting were selected to achieve enough resolution, high sensitivity, and high contrast ratio with the robustness against the environment change. XC-56 with VGA-class resolution (647 × 493) is a monochrome camera module in this research. It was mounted with a lens with C-mounting.

**Figure 2:**
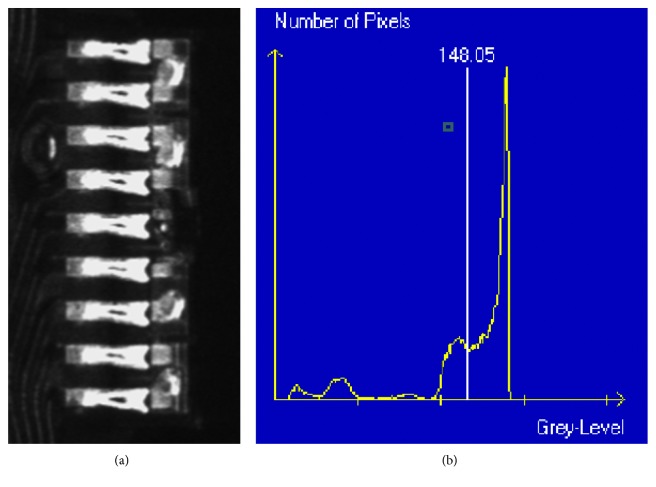
(a) Typical image of slider circuit on HGA. (b) Histogram of the typical image [3].

In this process, the threshold value was applied to the image transformation from grayscale image to binary image, and the value was specified based on considering the histogram at the preprocessing. As seen in Figure 2, the threshold value, 148.05, on the histogram can separate the group of pixel clearly to maximize the contrast of the captured image. The general image processing technique, i.e., noise elimination, “closing,” was also applied to the image preprocessing.

The feature extraction techniques were applied to find the candidate features on images. In this work, angle, area, inertia, center of mass, acircularity, perimeter, and elongation were determined. Examples of image feature calculation are shown in Equations (2) and (3), and these are area and acircularity, respectively:

(2) $$\begin{array}{c} A=\overset{N}{\underset{i=1}{\sum }}x_{i}y_{i}, \end{array}$$

(3) $$\begin{array}{c} C=\frac{P^{2}}{4\pi A}. \end{array}$$

To find the best features to be used in the clustering technique, the popular technique, “Branch and Bound (BNB)” was applied. The BNB process starts from the objective function, branches the big problem, and divides it into small group problems. Then, the process analyzes the bound of problem and removes some results, which cannot provide the best results evaluated from the objective function. The process is repeated until finding the best solution.

## 4. Clustering and the Proposed Technique

Classification is the method to classify data into particular groups by the model construction. All data are separated into 2 sets: training set and validation set. The training set is used for constructing the clustering structure and parameters, while the validation set is used for verifying the performance of the model. There are a number of techniques applied to clustering such as Euclidean distance. In this research, the performance of the proposed technique neuro-fuzzy c-means was investigated in comparison with those from the K-means and Kohonen clustering techniques. The following section describes the brief concept of each clustering technique applied in this research work.

### 4.1. K-Means Clustering

**Figure 3:**
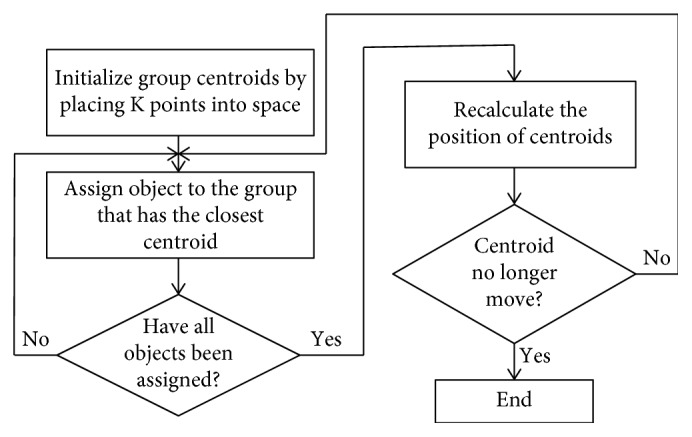
K-means clustering.

The K-means clustering technique defines the K value to represent the group member of the cluster, and the centroid value on each cluster is set for initial value. A point that shows the minimum summation distance between members and the centroid will be set as the center of that group. The process will be repeated and calculated to perform the new centroid on each cluster until the centroid value is not changed (convergence).  Figure 3 shows the steps of the K-means clustering technique.

The Euclidean distance as shown in (4) is used to calculate the K values and the centroid cluster as shown in (5):

(4) $$\begin{array}{c} \text{Min}\left[D\left(c_{i},x_{i}\right)=\sqrt{\overset{n}{\underset{i=1}{\sum }}{\left(c_{i}{\;-\;x}_{i}\right)}^{2}}\right], \end{array}$$

(5) $$\begin{array}{c} c_{i}=\frac{1}{\left|S_{i}\right|}\underset{x_{i}\in S_{i}}{\sum }x_{i}. \end{array}$$

where *S* is the total data point, *c*_*i*_ is the centroid cluster, and *x*_*i*_ is the data point.

### 4.2. Kohonen Clustering

**Figure 4:**
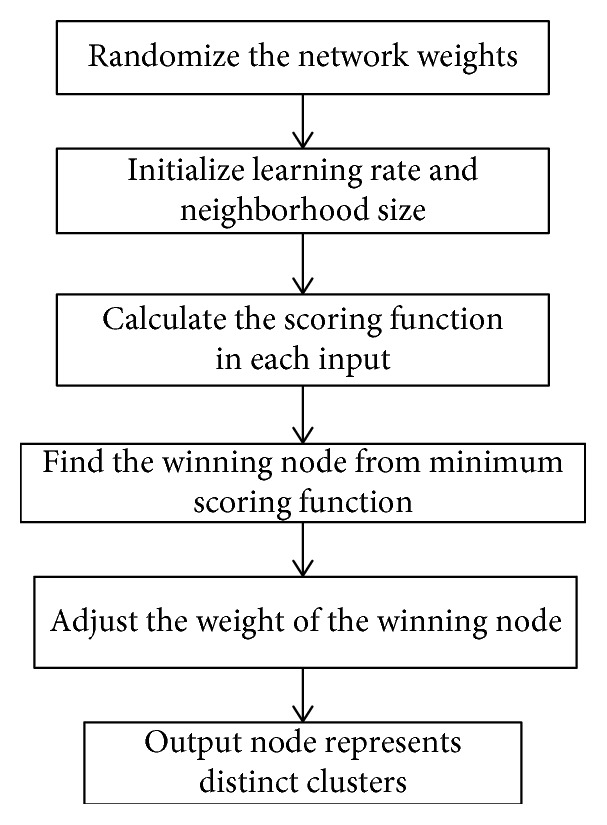
Kohonen clustering.

The Kohonen clustering starts by assigning the weight randomly and defines the learning rate and the dataset. The minimum distance is the winning node, and then, a new weight will represent each cluster. The new weight is calculated using the following equation:

(6) $$\begin{array}{c} w_{ij,\text{NEW}}=w_{ij,\text{CURRENT}}+\eta \left(x_{ni}-w_{ij,\text{CURRENT}}\right), \end{array}$$

where *w*_*ij*,NEW_ is the new weight matrix, *w*_*ij*,CURRENT_ is the current weight matrix, *x*_*ni*_ is the *n*^th^ data, and *η* is the learning rate. The updating calculation is repeated until the convergence condition is met. The steps of Kohonen mentioned above are shown in Figure 4.

### 4.3. Neurofuzzy c-Means Clustering

The neurofuzzy c-means clustering starts with an initial value and updates this value until achieving the stopping condition. The objective function in (7) is used for finding the optimal distance value (smallest value):

(7) $$\begin{array}{c} J_{\text{FCM}}=\overset{N}{\underset{i=1}{\sum }}\overset{C}{\underset{j=1}{\sum }}u_{ij}^{m}{\left\|x_{i}-c_{j}\right\|}^{2}, \end{array}$$

where *u*_*ij*_ is the membership function, *N* is the number of data, *c*_*j*_ is the centroid of the clustering, and *x*_*i*_ is the *i*th data. The membership function can be calculated from the following equation:

(8) $$\begin{array}{c} u_{ij}=\frac{1}{\sum_{k=1}^{C}{\left(\left\|x_{i}-c_{j}\right\|/\left\|x_{i}-c_{k}\right\|\right)}^{\left(2/\left(m-1\right)\right)}}. \end{array}$$

Clustering centers are given by the normalized and defuzzification technique (8):

(9) $$\begin{array}{c} c_{j}=\frac{\sum_{i=1}^{N}u_{ij}^{m}\cdot x_{i}}{\sum_{i=1}^{N}u_{ij}^{m}}. \end{array}$$

**Figure 5:**
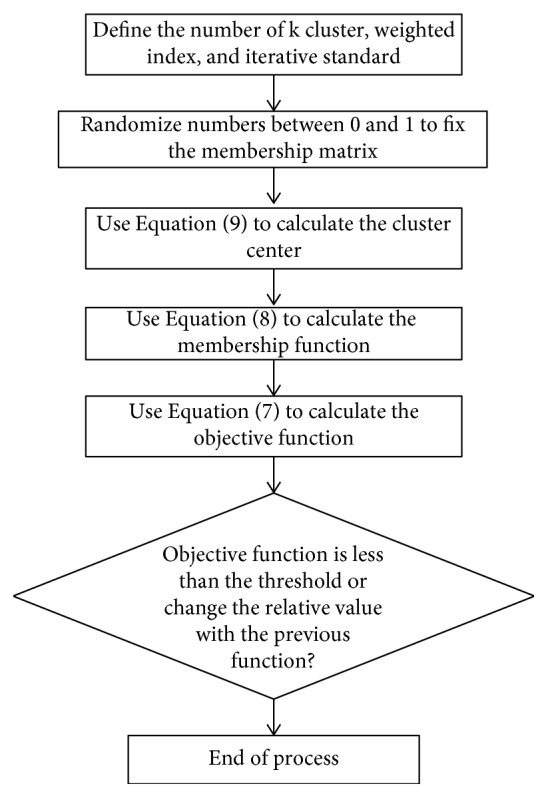
Neurofuzzy c-means clustering.

The steps of the neurofuzzy c-means clustering are shown in Figure 5. As seen in this figure, the objective function has been minimized to achieve the optimal values of the clustering center.

## 5. System Preparation and Experimental Results

**Figure 6:**
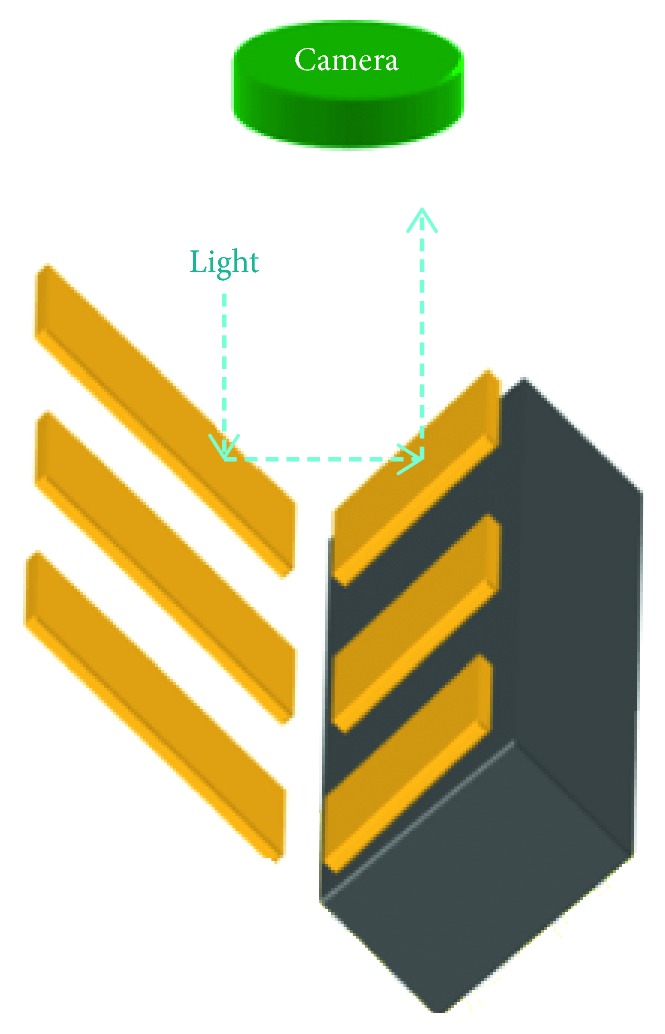
Visual inspection system on HGA circuit.

The lighting installation system is applied to illuminate the slider circuit and make the best contrast image of the HGA circuit, as shown in Figure 6. The selected type of lighting technique is the diffusion illumination.

The element on the HGA circuit was made from Au (gold). Since the slider is considered as the curve surface with medium reflection, the diffusion lighting technique was applied on this experiment with the ring light (LED) that is suitable because of its long lifetime, flexible application, and high brightness. Lens and camera selection were done by considering the field of view and depth of field. The HGA circuit area is 45 × 64 *µ*m (280 × 210), and the depth of field is 80 *µ*m. The selected camera is XC-56, having the total pixel as 659 × 464. Thus, the magnification of the lens can be calculated using (1) as

(10) $$\begin{array}{c} m=\frac{659\times 464}{280\times 210}=5.20. \end{array}$$

**Figure 7:**
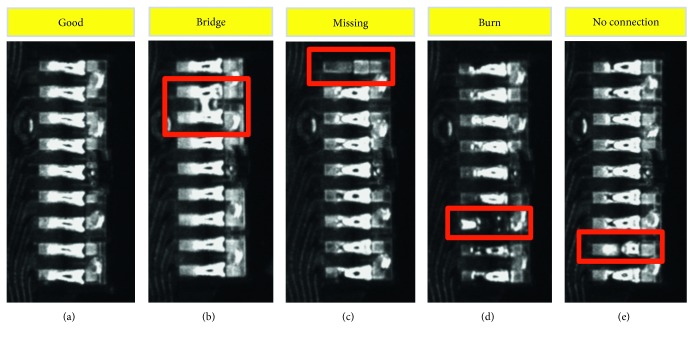
Image of HGA circuit in 5 groups: (a) good, (b) bridging, (c) missing, (d) burn, and (e) no connection.

The lens Infinitube FM-75 PL-18 Series with working distance 15 mm was selected. The 5 groups of HGA circuit classification are “good,” “bridging,” “missing,” “burn,” and “no connection” as shown in Figure 7.

**Figure 8:**
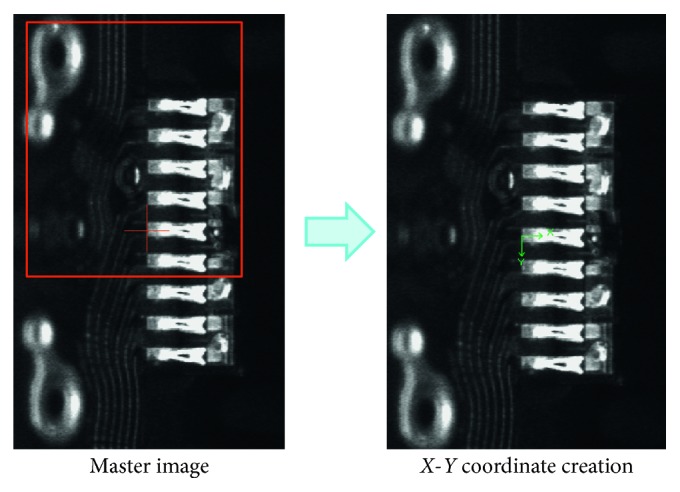
Master image and *X*, *Y* coordinate creation.

Cognex Vision Pro Version 7.2 was applied as the image processing tool, and the master image from the Seagate Company is used for training the neurofuzzy c-means for reference searching (Figure 8).

After the above process, the blob tool is used to separate the region of interest (ROI) and the background with the threshold value. As shown in Figure 2, 40% of the pixels are on the left-hand side, and 60% are on the right-hand side, which can effectively be separated by the threshold value. By using this relative threshold value, the vision process robustness is gained when the lighting changes.

**Figure 9:**
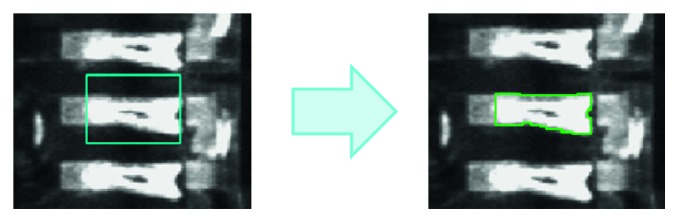
Image on blob processing.

After preprocessing and blob exporting, 8 image feature variables, which are area, moment of inertia, perimeter, acircularity, center of mass X, center of mass Y, elongation, and angle, were exported.  Figure 9 shows preprocessing to define the ROI and using blob to find the interesting features. The objective function in the proposed technique to be utilized in BNB is shown in the following equation:

(11) $$\begin{array}{c} \text{objective function}=\frac{\text{count of correct class index}}{\text{total data}}*100. \end{array}$$

**Figure 10:**
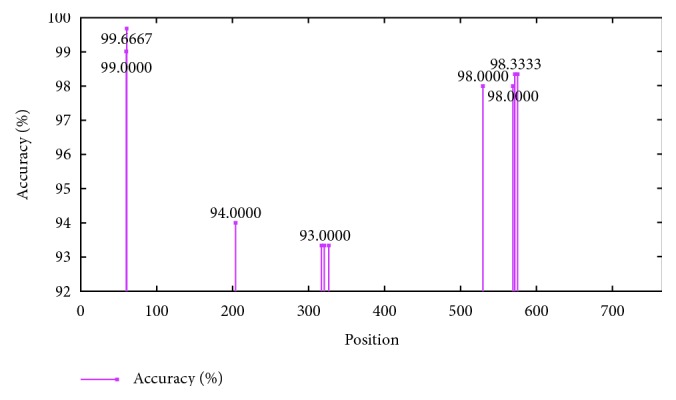
Top 10 of best accuracy value chart.

Objective function is defined as the percentage of the accuracy when comparing with the results by human eyes classification using the 30x microscope. The event that obtains the best value (highest objective function value) is the best classification structure for this application. Three clustering techniques were applied to the system which are used to calculate the accuracy sets to find the best classification technique and best features. Through the BNB technique with the 3 classification techniques, 8 image features, 5 classification groups, and 300 samples, the optimal centroid values for 5 predefined classification groups were evaluated. The results show the best accuracy of 99.667% by the neurofuzzy c-means clustering.  Figure 10 and Table 1 illustrate the details of the results.

Table 1 shows the top 10 of best accuracy and their details.

The best accuracy can be achieved by the NFC technique, and the 3 image feature variables are area, moment of inertia, and perimeter. Clearly, the design using the neurofuzzy c-means achieves the best performance and can be adopted to command the SCARA robot to classify the quality of the product after slider attachment process.

**Figure 11:**
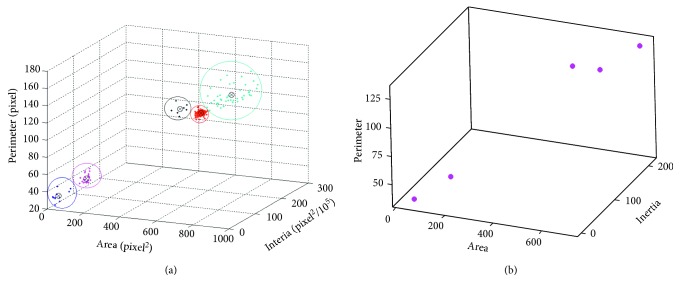
Clustering results of best accuracy (61st): (a) clustering groups and data and (b) centroid of each clustering group.

As shown in Figure 11(a), the red circle is the group of “Good,” the blue circle is “Bridging,” the pink circle is “Missing,” the dark blue circle is “Burn,” and the black circle is “No connection.” Figure 11(b) shows the centroid of each group, and Table 2 shows the optimal centroid of each group.

**Figure 12:**
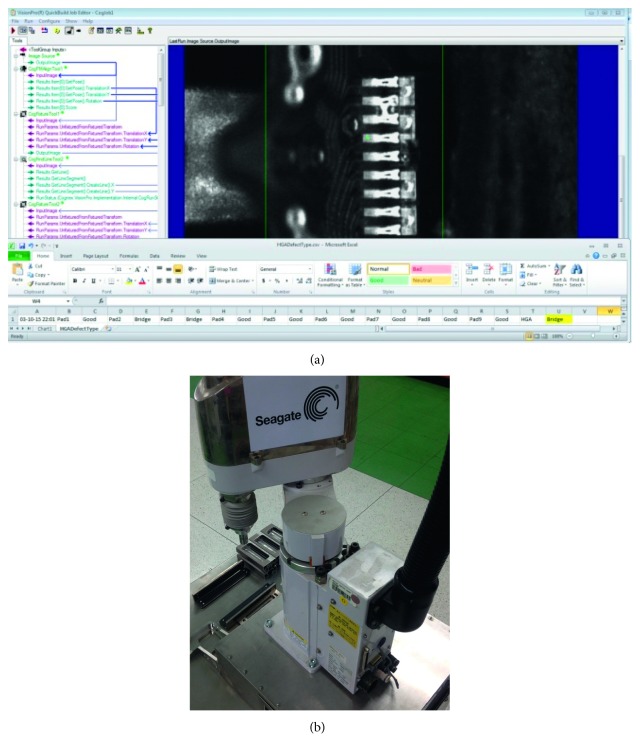
(a) Image processing and CSV file exporting and (b) cleanroom type SCARA robot used in the slider movement machine.

In the validation process, the selected variables, which are area, moment of inertia, and perimeter, with NFC clustering were adopted. 300 new samples were tested on this validation process, and 3 visual inspection machines, machine no. 17, no. 23, and no. 101, were used to perform the test to ensure the performance on different machines using the same technique. 100 samples per each machine were tested, and it was found that the accuracy achieved on machine no. 17, 23, and 101 were 100%, 99.21%, and 99.12%, respectively. Clearly, the above results show the accuracy more than 99% (achieved the Seagate's criteria) for the 3 vision systems, and the technique can be implemented in the actual production line. The example of image construction and real implementation in production line, clustering by NFC, is shown in Figure 12. Also, the SCARA robot in the machine which is used to transfer the slider based on the evaluation results of the NFC is shown in Figure 12(b).

## 6. Conclusion

As shown in the experiment results, the excellent performance with 99.67% accuracy can be achieved from the neurofuzzy c-means clustering with three best features of area, moment of inertia, and perimeter in the training process. The accuracy was investigated by comparing with the results from the 30x microscope inspection using human operations. In the validation process, the accuracy of more than 99% with the 3 different vision systems can be achieved. This confirms that the proposed method can be applied to the inspection of the Head Gimbal Assembly circuit classification. In addition, the command to move the slider works well with the SCARA robot to move the HGA stack to the classified boxes correctly.

## Figures and Tables

**Table 1:** Top 10 of best accuracy and details.

Order	Axis	Clustering	Best variable
1 (61st)	3	NFC	Area, moment of inertia, perimeter
2 (60th)	3	K-means	Area, moment of inertia, acircularity
3 (517th)	3	NFC	Area, moment of inertia ,perimeter
4 (575th)	3	NFC	Area, center of mass X, perimeter
5 (529th)	2	NFC	Area, acircularity
6 (568th)	3	NFC	Area, moment of inertia, center of mass X
7 (204th)	5	K-means	Area, moment of inertia, center of mass Y, acircularity, perimeter
8 (317th)	3	Kohonen	Area, moment of inertia, elongation
9 (321st)	3	Kohonen	Area, center of mass X, elongation
10 (327th)	3	Kohonen	Area, perimeter, elongation

**Table 2:** Centroid values of 5 groups from the best clustering results (61st clustering structure).

Group	Area (pixel^2^)	Moment of inertia (pixel^2^/10^5^)	Perimeter (pixel)
Good	556.7256	200.5483	108.4784
Bridging	708.2615	211.1732	131.5751
Missing	198.7688	11.3017	57.9568
Burn	60.4703	1.8718	36.1733
No connection	474.0023	178.3713	115.4271

## Data Availability

The images from the SCARA robot in this article were analyzed in the proposed algorithm which is shown in numerical data as double type. All numerical data are the output from the vision algorithm and tested in the clustering algorithm in the MATLAB program. This research used the image from the SCARA robot for analysis which is included in the article and as supplied by Seagate Technology (Thailand) Co., Ltd. and so cannot be made freely available. Request for access to these data should be made to Veerasak Phana-ngam via mail (veerasak.phana-ngam@seagate.com).
